# CRISPR/Cas9‐mediated genome editing reveals seven testis‐enriched transmembrane glycoproteins dispensable for male fertility in mice

**DOI:** 10.1111/andr.13564

**Published:** 2023-12-12

**Authors:** Yo Ogawa, Yonggang Lu, Daiji Kiyozumi, Hsin‐Yi Chang, Masahito Ikawa

**Affiliations:** ^1^ Graduate School of Pharmaceutical Sciences Osaka University Suita Japan; ^2^ Department of Experimental Genome Research, Research Institute for Microbial Diseases Osaka University Suita Japan; ^3^ Premium Research Institute for Human Metaverse Medicine (WPI‐PRIMe) Osaka University Suita Japan; ^4^ Research Institute of Environmental Medicine Nagoya University Nagoya Japan; ^5^ Graduate School of Medicine Osaka University Suita Japan; ^6^ The Institute of Medical Science The University of Tokyo Tokyo Japan

**Keywords:** CRISPR/Cas9, fertilization, male fertility, sperm morphology, sperm motility, spermatogenesis

## Abstract

**Background:**

Mammalian fertilization is mediated by multiple sperm acrosomal proteins, many of which are testis‐enriched transmembrane glycoproteins expressed during spermiogenesis (e.g., Izumo sperm‐egg fusion 1, Sperm acrosome associated 6, and Transmembrane protein 95).

**Methods:**

We hypothesized that proteins with these features might have a role in sperm–egg interaction and thus carried out an in‐silico screen based on multiple public databases. We generated knockout mouse lines lacking seven candidate proteins by the CRISPR/Cas9 system and conducted detailed analyses on the fecundity of the knockout males, as well as their testis appearance and weight, testis and epididymis histology, and sperm motility and morphology.

**Results:**

Through the in‐silico screen, we identified 4932438H23Rik, A disintegrin and metalloproteinase domain‐containing protein 29, SAYSvFN domain‐containing protein 1, Sel‐1 suppressor of lin‐12‐like 2 (*C. elegans*), Testis‐expressed protein 2, Transmembrane and immunoglobulin domain‐containing 3, and Zinc and ring finger 4. Phenotypic analyses unveiled that the knockout males showed normal testis gross appearance, normal testis and epididymis histology, and normal sperm morphology and motility. Fertility tests further indicated that the knockout male mice could sire pups with normal litter sizes when paired with wild‐type females.

**Discussion and conclusion:**

These findings suggest that these seven proteins are individually dispensable for male reproduction and fertilization. Future studies are warranted to devise advanced in‐silico screening approaches that permit effective identification of gamete fusion‐required sperm proteins.

## 1 INTRODUCTION

During mammalian reproduction, spermatozoa travel through the female reproductive tract and undergo a series of biochemical and physiological changes, collectively known as capacitation, to acquire fertilization ability.^1^, ^2^ Before approaching an egg, a  spermatozoon elicits the acrosome reaction, where the sperm head plasma membrane fuses with the acrosomal membrane to expose the acrosomal contents such as hydrolytic enzymes and acrosomal membrane proteins.^3^ The enzymes allow spermatozoa to penetrate the cumulus cell layer and the zona pellucida (ZP),^4^ whereas multiple acrosomal membrane proteins regulate sperm–egg interaction.^5^

We previously reported that Izumo sperm–egg fusion 1 (IZUMO1), Sperm acrosome associated 6 (SPACA6), Fertilization influencing membrane protein (FIMP), Transmembrane protein 95 (TMEM95), Sperm‐oocyte fusion‐required 1 (SOF1), Dendritic cell‐specific transmembrane protein domain‐containing protein 1/2 (DCST1/2), and TMEM81 are involved in sperm–egg membrane binding or fusion.^6^, ^7^, ^8^, ^9^, ^10^, ^11^ However, none of these fusion‐related elements in gametes (FREGs) show structural resemblance to known fusogens or demonstrate fusogenic activity in vivo. Interestingly, SPACA6 is absent in mouse spermatozoa lacking any one of the known sperm FREGs; yet, whether sperm FREGs form a protein complex to orchestrate plasma membrane fusion between spermatozoa and eggs remains to be explored.^11^ To better understand the underlying molecular mechanisms, it is crucial to discover more proteins essential for sperm–egg interaction. Among the known sperm FREGs, IZUMO1, SPACA6, TMEM95, and DCST1/2 are testis‐specific, *N*‐linked glycosylated, and predominantly expressed in round spermatids^12^; these common features might serve as criteria for discovering additional sperm FREGs involved in fertilization.

CRISPR/Cas9 is a powerful genome editing technique that has contributed to the discovery of many essential male reproductive factors, such as Leucine‐rich repeat containing 23 (LRRC23), a radial spoke protein necessary for sperm motility,^13^ and Neural EGF‐like like 2 (NELL2) and NELL2‐interacting cofactor for lumicrine signaling (NICOL), testis‐derived secreted proteins required for epididymal and sperm maturation.^14^, ^15^ In this study, we surveyed multiple public databases for genes encoding transmembrane glycoproteins exclusively expressed during spermiogenesis and generated knockout (KO) mouse lines lacking each gene by CRISPR/Cas9 to interrogate their essentiality for male reproduction and fertilization.

## 2 MATERIALS AND METHODS

### 2.1 Animals

All mice were maintained under a specific‐pathogen‐free condition with ad libitum feeding. All experiments involving animals were approved by the Institutional Animal Care and Use Committee of Osaka University in compliance with relevant guidelines and regulations. In this study, all B6D2F1/J and ICR mice were purchased from Japan SLC. All mutant mouse lines were generated on a hybrid genetic background of B6D2F1/J and the frozen spermatozoa of heterozygous KO mice were deposited to RIKEN BioResource Research Center (web.brc.riken.jp/en) and Center for Animal Resources and Development at Kumamoto University (card.medic.kumamoto‐u.ac.jp/card/english; Table S1).

### 2.2 Generation of KO mice by CRISPR/Cas9

All KO mouse lines were generated by the CRISPR/Cas9 genome editing system as previously described.^16^ Single‐guide RNAs (sgRNAs) were designed (Table S2) using the online software CRISPRdirect (crispr.dbcls.jp), CRISPOR (crispor.org), and Benchling (benchling.com). The ribonucleoprotein complexes containing CRISPR RNA (crRNA; Sigma‐Aldrich), trans‐activating crRNA (tracrRNA; Thermo Fisher Scientific), and Cas9 protein (Thermo Fisher Scientific) were introduced into zygotes obtained from B6D2F1/J females crossed with B6D2F1/J males using a Nepa Gene NEPA21 electroporator. The embryos were transferred to the oviductal ampullae in ICR pseudopregnant females and the founder offspring were obtained by natural delivery or Caesarean section. The mutant alleles were identified by genomic PCR and subjected to Sanger sequencing. The sequences of sgRNAs and primers and PCR conditions are listed in Tables S2 and S3.

### 2.3 Fertility tests

Sexually mature B6D2F1/J (wild‐type [WT]) or KO male mice were caged individually with three 6‐week‐old WT female mice. After 8 weeks of natural mating, the male mice were removed from the cages, and the females were kept for another 3 weeks to deliver their final litters. During the fertility tests, the number of pups and copulation plugs were recorded.

### 2.4 Testis histology, sperm morphology, and sperm motility

After sexual maturation, three KO male mice and three same‐aged heterozygous littermates or WT male mice were euthanized and their testes and epididymides were isolated. Spermatozoa were extracted from cauda epididymides, dispersed in the Toyoda, Yokoyama, and Hoshi (TYH) medium,^17^, ^18^ and incubated for 10 min at 37°C, 5% CO_2_. Sperm morphology was observed under an Olympus BX53 differential interference contrast microscope. Sperm motility was measured using the Hamilton Thorne CEROS II sperm analysis system at 10 and 120 min of incubation in the TYH medium. Testes and epididymides were fixed in Bouin's fluid (Polysciences), embedded in paraffin wax, sectioned at a thickness of 5 μm on a Leica Microm HM325 rotary microtome, and stained with 1% periodic acid (Nacalai Tesque) and Schiff's reagent (Wako), followed by counterstaining with Mayer's hematoxylin solution (Wako).

### 2.5 Statistical analysis

The statistical difference was determined by the Student's t‐test. Differences were considered to be statistically significant if the *P* value was less than 0.05. Error bars represent the standard deviation.

## 3 RESULTS

### 3.1 In‐silico screen of transmembrane glycoproteins expressed in early spermatids

**FIGURE 1:**
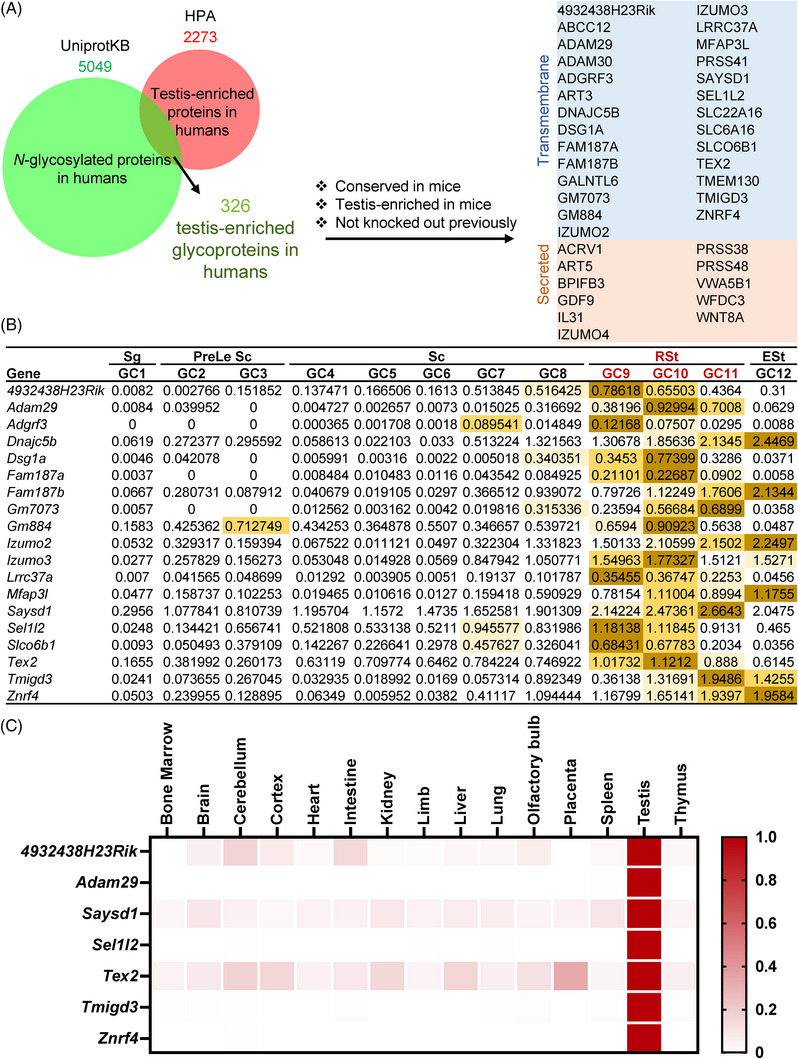
In‐silico screen of testis‐enriched transmembrane glycoproteins. (A) Schematic overview of the in‐silico screen. (B) The expression patterns of 19 transmembrane glycoproteins during spermatogenesis depicted by a published single‐cell RNA sequencing (scRNA‐seq) analysis. In each row, the spermatogenic cell stages with the top three expression levels have been highlighted in brown, yellow, and cream. GC, germ cell cluster; Sg, spermatogonia; PreLe Sc, preleptotene spermatocyte; Sc, spermatocyte; RSt, round spermatid; ESt, elongating spermatid. (C) Tissue expression patterns of the seven genes analyzed in this study. Data were retrieved from the Mouse ENCODE Project. The expression level in each tissue has been normalized to the RPKM (reads per kilobase of exon per million reads mapped) value of testis expression.

We obtained 5049 entries of *N*‐linked glycosylated proteins in humans through a keyword search in the Uniprot Knowledgebase (UniProtKB; uniprot.org) and 2273 entries of testis‐enriched proteins in humans from Human Protein Atlas (HPA; proteinatlas.org). A Venn diagram analysis revealed 326 overlapped proteins that are potentially *N*‐glycosylated and highly expressed in the human testis (Figure 1A). We then subtracted proteins that are not conserved in mice, do not show testis‐enriched expression in mice, or have been previously knocked out in mice by referring to the NCBI database (ncbi.nlm.nih.gov), mouse ENCODE transcriptome database (encodeproject.org), and Mouse Genome Informatics database (informatics.jax.org), respectively. The resultant 38 entries include 11 secreted proteins and 27 transmembrane proteins (Figure 1A). We further analyzed the mRNA expression of the 27 transmembrane proteins during spermatogenesis by referring to previously published single‐cell RNA sequencing datasets.^19^ As a result, 19 genes were found to exhibit biased expression in round or elongating spermatids (Figure 1B). We selected seven genes, *4932438H23Rik*, *Adam29*, *Saysd1*, *Sel1l2*, *Tex2*, *Tmigd3*, and *Znrf4*, for further analyses (Figure 1C).

### 3.2 Generation and phenotypic analyses of KO mouse lines

Individual gene KO mouse lines were generated by CRISPR/Cas9 (see **Materials and Methods**), and phenotypic analyses were conducted to investigate the essentiality of these genes in male reproduction. The analyses of *Saysd1*, *Tmigd3*, and *Znrf4* KO males will be described herein, whereas those of *4932438H23Rik*, *Adam29*, *Sel1l2*, and *Tex2* KO males are presented in Figures S1–S4.

**FIGURE 2:**
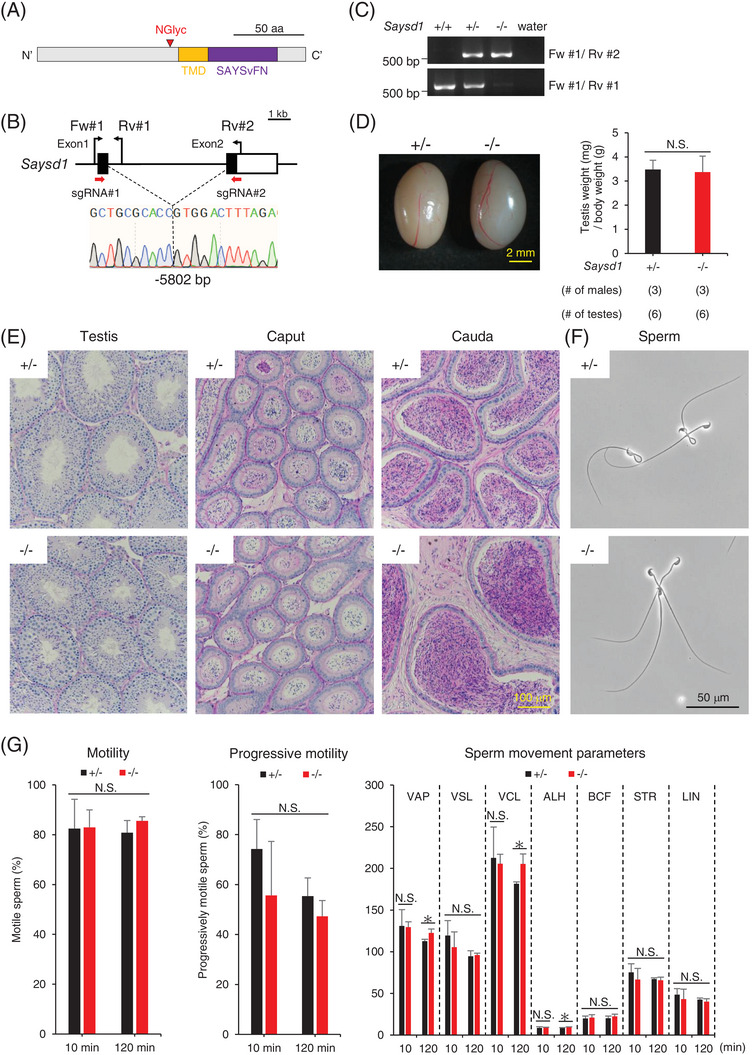
Phenotypic analysis of *Saysd1* knockout (KO) male mice. (A) Domain composition of mouse SAYSD1. The red arrowhead, the orange box, and the purple box indicate an *N*‐glycosylation site (NGlyc), a transmembrane domain (TMD), and an SAYSvFN motif, respectively. (B) Genomic structure and KO strategy of mouse *Saysd1*. Fw #1, forward primer for genotyping; Rv #1/#2, reverse primers for genotyping. (C) Genotypic validation of *Saysd1* mutant mice. (D) Testis gross appearance and relative weight (normalized to the body weight) in *Saysd1* ^+/−^ and *Saysd1* ^−/−^ mice. (E) Histology of testes and epididymides in *Saysd1* ^+/−^ and *Saysd1* ^−/−^ mice. (F) Morphology of cauda epididymal spermatozoa in *Saysd1* ^+/−^ and *Saysd1* ^−/−^ mice. (G) Motility of cauda epididymal spermatozoa in *Saysd1* ^+/−^ and *Saysd1* ^−/−^ mice. Sperm motility and kinetic parameters were measured after incubation in TYH media for 10 and 120 min. VAP, average path velocity; VSL, straight‐line velocity; VCL, curvilinear velocity; ALH, amplitude of lateral head displacement; BCF, beat cross frequency; STR, straightness; LIN, linearity.

*Saysd1* is a reverse‐strand gene localized to mouse chromosome 14. It encodes a type I transmembrane protein carrying an *N*‐glycosylation site and an SAYSvFN motif with no defined function (Figure 2A). Using two sgRNAs targeting the 5′ and 3′ regions of *Saysd1*, we obtained mutant mice harboring a 5802 bp deletion (Figure 2B,C). *Saysd1* ^+/−^ and *Saysd1* ^−/−^ males showed normal testis gross appearance and comparable testis weights relative to the body weights (Figure 2D). Histological analyses revealed no abnormality in the histology of testes and epididymides in *Saysd1* ^+/−^ and *Saysd1* ^−/−^ males (Figure 2E). Furthermore, *Saysd1* ^+/−^ and *Saysd1* ^−/−^ males exhibited normal sperm morphology (Figure 2F). Computer‐assisted sperm analysis (CASA) indicated that sperm motility, progressive motility, and sperm swimming velocity were comparable between *Saysd1* ^+/−^ and *Saysd1* ^−/−^ males (Figure 2G).

**FIGURE 3:**
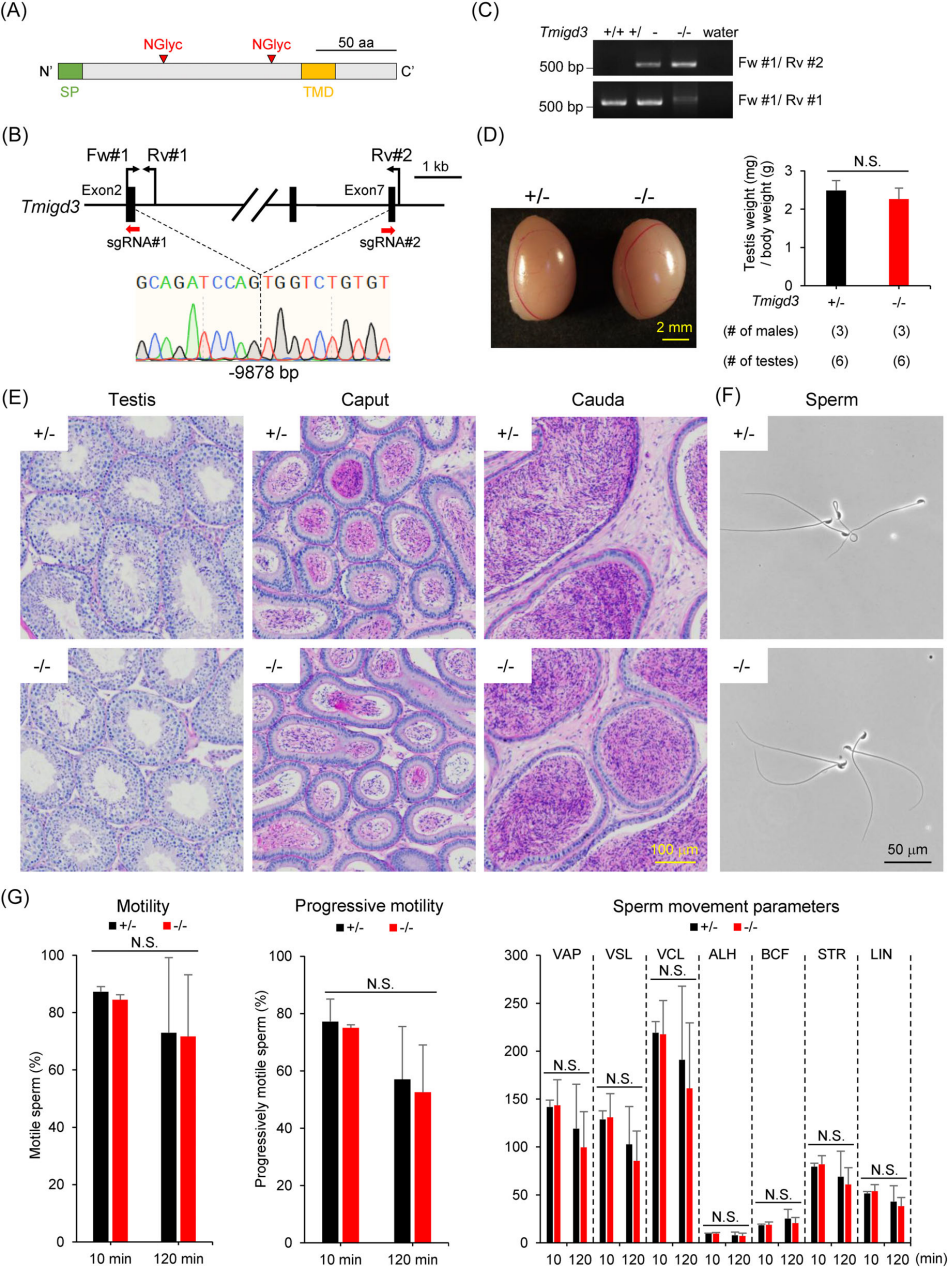
Phenotypic analysis of *Tmigd3* knockout (KO) male mice. (A) Domain composition of mouse TMIGD3 protein. The green box represents a signal peptide. (B) Genomic structure and KO strategy of mouse *Tmigd3*. (C) Genotypic validation of *Tmigd3* mutant mice. (D) Testis gross appearance and relative weight (normalized to the body weight) in *Tmigd3* ^+/−^ and *Tmigd3* ^−/−^ mice. (E) Histology of testes and epididymides in *Tmigd3* ^+/−^ and *Tmigd3* ^−/−^ mice. (F) Morphology of cauda epididymal spermatozoa in *Tmigd3* ^+/−^ and *Tmigd3* ^−/−^ mice. (G) Motility of cauda epididymal spermatozoa in *Tmigd3* ^+/−^ and *Tmigd3* ^−/−^ mice. Sperm motility and kinetic parameters were measured after incubation in TYH media for 10 and 120 min.

TMIGD3 is a type I transmembrane protein with two *N*‐glycosylation sites (Figure 3A). This protein is encoded by a gene located on the forward strand of mouse chromosome 3. Similarly, *Tmigd3* was knocked out by targeting its coding region using two sgRNAs, and mutant offspring bearing a 9878‐bp deletion were identified by genomic PCR and Sanger sequencing (Figure 3B,C). Phenotypic analyses revealed that *Tmigd3* ^−/−^ males showed normal testicular appearance and weights (Figure 3D), normal testicular and epididymal histology (Figure 3E), and normal sperm morphology (Figure 3F) and motility (Figure 3G).

**FIGURE 4:**
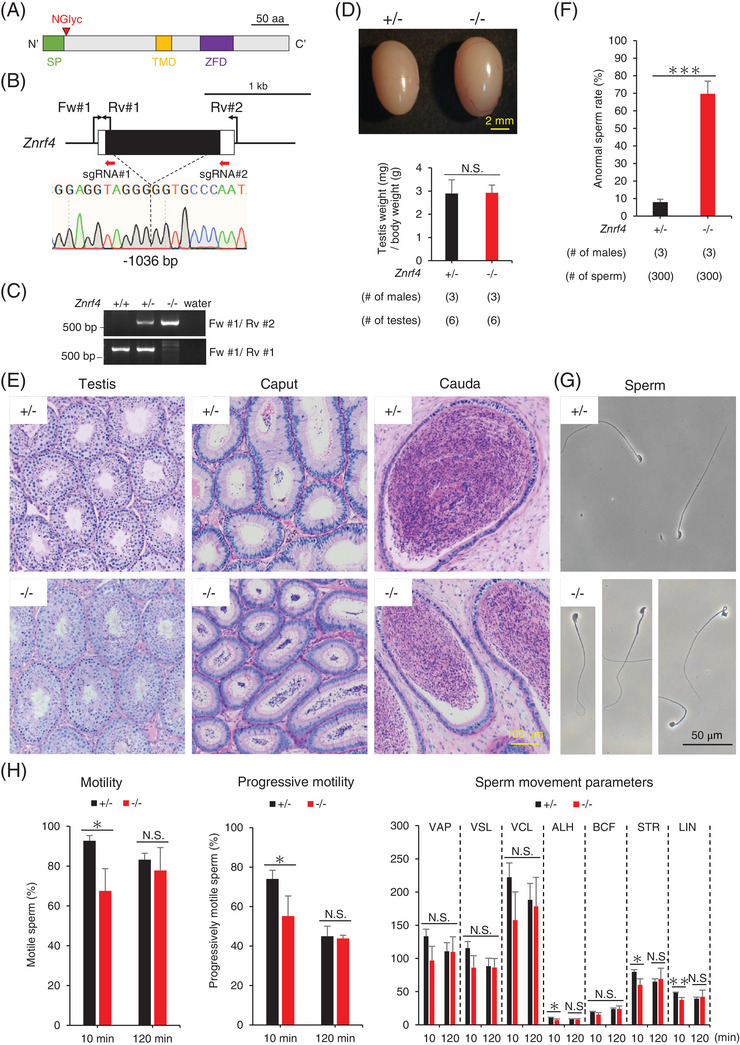
Phenotypic analysis of *Znrf4* knockout (KO) male mice. (A) Domain composition of mouse ZNRF4. The purple box indicates a zinc finger domain (ZFD). (B) Genomic structure and KO strategy of mouse *Znrf4*. (C) Genotypic validation of *Znrf4* mutant mice. (D) Testis gross appearance and relative weight (normalized to the body weight) in *Znrf4* ^+/−^ and *Znrf4* ^−/−^ mice. (E) Histology of testes and epididymides in *Znrf4* ^+/−^ and *Znrf4* ^−/−^ mice. (F–G) Morphology of cauda epididymal spermatozoa in *Znrf4* ^+/−^ and *Znrf4* ^−/−^ mice. (H) Motility of cauda epididymal spermatozoa in *Znrf4* ^+/−^ and *Znrf4* ^−/−^ mice. Sperm motility and kinetic parameters were measured after incubation in TYH media for 10 and 120 min.

*Znrf4* is a single‐exon gene localized to the reverse strand of mouse chromosome 17. It encodes a type I transmembrane protein carrying an *N*‐glycosylation site and a RING‐type zinc finger domain (Figure 4A). Using two sgRNAs flanking the single exon, we introduced a 1036 bp deletion in the locus of *Znrf4* (Figure 4B,C). *Znrf4* ^−/−^ mice showed normal testis appearance and weights (Figure 4D) and normal testicular and epididymal histology (Figure 4E). Noticeably, nearly 70% of *Znrf4* KO spermatozoa exhibited morphological abnormalities, such as bent sperm heads, loss of apical hook, and irregular‐shaped sperm heads (Figure 4F,G). The motility of *Znrf4* KO spermatozoa was comparable with that of the control spermatozoa (Figure 4H).

### 3.3 Fertility tests for KO male mice

By housing the KO males individually with three WT females for 8 weeks, we discovered that the males could sire offspring with average litter sizes comparable to those of WT males (Table 1).

**TABLE 1:** Fertility tests for male mice lacking the seven genes. Wild‐type (WT) males were tested in parallel as a positive control.

Gene symbol	Genotype	No. of males	No. of pups	No. of litters	Mating period	Average litter size ± SD
**WT**	+/+	3	247	30	8 weeks	8.23 ± 2.96
***4932438H23Rik***	−678+5/−678+5	3	226	26	8 weeks	8.31 ± 2.57
***Adam29***	−2448/−2448	3	190	24	8 weeks	8.40 ± 1.31
***Saysd1***	−5802/−5802	3	256	26	8 weeks	9.85 ± 1.87
***Sel1l2***	−159716/−159716	3	234	25	8 weeks	9.36 ± 3.24
***Tex2***	−65130/−65130	3	228	23	8 weeks	9.91 ± 3.33
***Tmigd3***	−9878/−9878	3	216	23	8 weeks	9.39 ± 2.13
***Znrf4***	−1036/−1036	3	106	15	8 weeks	7.07 ± 1.98

## 4 DISCUSSION

We previously discovered that multiple sperm FREGs, including IZUMO1, SPACA6, TMEM95, and DCST1/2, are transmembrane glycoproteins predominantly expressed in round spermatids.^19^ In search of more proteins harboring these features, we performed an in‐silico screen and identified 4932438H23Rik, ADAM29, SAYSD1, SEL1L2, TEX2, TMIGD3, and ZNRF4. The KO male mice lacking any one of these proteins exhibited normal fecundity, suggesting that these proteins are individually dispensable for male reproduction and fertilization.

ZNRF4 contains a RING‐type zinc finger domain and reportedly functions as an E3‐ubiquitin ligase that attenuates immune response by degrading the NOD2 (Nucleotide‐binding oligomerization domain‐containing protein 2) signaling adaptor.^20^ A recent study indicates that ZNRF4 is downregulated in the spermatozoa from human patients carrying biallelic mutations in *SPEF2* (Sperm flagellar 2).^21^ *SPEF2* mutant male patients show severe asthenoteratozoospermia due to defective spermiogenesis. *Spef2* KO mice show abnormal sperm tail morphology and abnormal sperm head shape, indicating the potential roles of SPEF2 in sperm development and morphogenesis.^22^, ^23^ As the downregulation of ZNRF4 might originate from lower sperm numbers or impaired sperm structure in *SPEF2* mutant patients, further investigation is warranted to uncover the physiological function of ZNRF4. Despite that the majority of *Znrf4* KO spermatozoa are morphologically abnormal, our fertility tests suggest that the remaining normal spermatozoa are sufficient to fertilize eggs in vivo.

ADMA29 is a member of the ADAM family proteins and is highly expressed in human and mouse testis.^24^, ^25^ ADAM proteins are characterized by their metalloprotease and integrin‐receptor‐binding activities and most of them are expressed mainly in the male reproductive tissues.^26^, ^27^ Several ADAM proteins such as ADAM2 and ADAM3 have been reported to play important roles in sperm oviductal migration and sperm–ZP binding in mice.^28^, ^29^, ^30^, ^31^ ADAM29 has several homologous proteins such as ADAM24, ADAM25, and ADAM26 (Figure S5A), which are also predominantly expressed in post‐meiotic male germ cells.^26^, ^32^, ^33^ It has been reported that *Adam24* KO mice show reduced fecundity due to polyspermic fertilization.^34^ ADAM29 is downregulated in human spermatozoa harboring a missense *TEX101* variant rs35033974.^35^ Thus, it was tempting to speculate that ADAM29, similar to several other ADAM family proteins (e.g., ADAM3 and ADAM7) and TEX101, facilitates sperm fertilization ability.^25^, ^30^, ^32^, ^33^ However, *Adam29* KO mice exhibited normal fertility, indicating that the depletion of ADMA29 alone does not impair the function of TEX101.

The proteins disrupted in this study are dispensable for male reproduction, suggesting that our in‐silico screen focusing on testis‐enriched transmembrane glycoproteins is inadequate for identifying sperm FREGs. Recent structural analyses indicate that both IZUMO1 and SPACA6 consist of a four α‐helix bundle, a central β‐hairpin, and an immunoglobulin (Ig)‐like domain in their ectodomains.^36^, ^37^, ^38^, ^39^ Similarly, TMEM95 harbors an N‐terminal coiled‐coil domain containing three α‐helices and a β‐hairpin but lacks an Ig‐like domain in its extracellular region.^40^ Given the structural similarities among these proteins, we envision that future screens based on the existing criteria and protein 3D structures might be feasible to discover more male factors required for sperm–egg interaction.^11^

## AUTHOR CONTRIBUTIONS

Yo Ogawa, Yonggang Lu, and Masahito Ikawa designed the research and wrote the paper; Yo Ogawa, Yonggang Lu, Daiji Kiyozumi, and Hsin‐Yi Chang performed experiments and analyzed the data.

## Supporting information

Supporting Information

Supporting Information
