# Supplementary material for: CRISPR/Cas9‐mediated genome editing reveals seven testis‐enriched transmembrane glycoproteins dispensable for male fertility in mice
Source: Andrology. 2023 Dec 12;13(5):1251–60. doi: 10.1111/andr.13564 (PMC11166886; doi:10.1111/andr.13564)
Supplement: Supplementary file 1 — Supporting Information [file ANDR-13-1251-s002.docx]

## Supplementary Figures

**Figure S1. Phenotypic analysis of *4932438H23Rik* KO male mice. (A)** Domain composition of mouse 4932438H23Rik. Noticeably, TMHMM2.0 predicts that the N-terminus of 4932438H23Rik (highlighted in green) is a transmembrane domain (services.healthtech.dtu.dk/services/TMHMM-2.0), whereas UniProtKB recognizes that region as a signal peptide. **(B)** Genomic structure and KO strategy of mouse *4932438H23Rik.* **(C)** Genotypic validation of *4932438H23Rik* mutant mice. **(D)** Testis gross appearance and relative weight (normalized to the body weight) in *4932438H23Rik*^+/-^ and *4932438H23Rik*^-/-^ mice. **(E)** Histology of testes and epididymides in *4932438H23Rik* ^+/-^ and *4932438H23Rik*^-/-^ mice. **(F)** Morphology of cauda epididymal sperm in *4932438H23Rik*^+/-^ and *4932438H23Rik*^-/-^ mice. **(G)** Motility of cauda epididymal sperm in *4932438H23Rik*^+/-^ and *4932438H23Rik*^-/-^ mice. Sperm motility and kinetic parameters were measured after incubation in TYH media for 10 and 120 min.

**Figure S2. Phenotypic analysis of *Adam29* KO male mice. (A)** Domain composition of mouse ADAM29. The purple box represents a disintegrin domain. **(B)** Genomic structure and KO strategy of mouse *Adam29.* **(C)** Genotypic validation of *Adam29* mutant mice. **(D)** Testis gross appearance and relative weight (normalized to the body weight) in *Adam29*^+/+^ and *Adam29*^-/-^ mice. **(E)** Histology of testes and epididymides in *Adam29*^+/+^ and *Adam29*^-/-^ mice. **(F)** Morphology of cauda epididymal sperm in *Adam29*^+/+^ and *Adam29*^-/-^ mice. **(G)** Motility of cauda epididymal sperm in *Adam29*^+/+^ and *Adam29*^-/-^ mice. Sperm motility and kinetic parameters were measured after incubation in TYH media for 10 and 120 min.

**Figure S3. Phenotypic analysis of *Sel1l2* KO male mice. (A)** Domain composition of mouse SEL1L2. **(B)** Genomic structure and KO strategy of mouse *Sel1l2.* **(C)** Genotypic validation of *Sel1l2* mutant mice. **(D)** Testis gross appearance and relative weight (normalized to the body weight) in *Sel1l2*^+/-^ and *Sel1l2*^-/-^ mice. **(E)** Histology of testes and epididymides in *Sel1l2*^+/-^ and *Sel1l2*^-/-^ mice. **(F)** Morphology of cauda epididymal sperm in *Sel1l2*^+/-^ and *Sel1l2*^-/-^ mice. **(G)** Motility of cauda epididymal sperm in *Sel1l2*^+/-^ and *Sel1l2*^-/-^ mice. Sperm motility and kinetic parameters were measured after incubation in TYH media for 10 and 120 min.

**Figure S4. Phenotypic analysis of *Tex2* KO male mice. (A)** Domain composition of mouse TEX2. The purple box indicates an SMP-LTD domain that binds various glycerophospholipids. **(B)** Genomic structure, KO strategy and genotype validation by Sanger sequencing of mouse *Tex2*. **(C)** Genotype validation of *Tex2* KO mice by genomic PCR. **(D)** Testis gross appearance and relative weight (normalized to the body weight) in *Tex2*^+/-^ and *Tex2*^-/-^ mice. **(E)** Histology of testes and epididymides in *Tex2*^+/-^ and *Tex2*^-/-^ mice. **(F)** Morphology of cauda epididymal sperm in *Tex2*^+/-^ and *Tex2*^-/-^ mice. **(G)** Motility of cauda epididymal sperm in *Tex2*^+/-^ and *Tex2*^-/-^ mice. Sperm motility and kinetic parameters were measured after incubation in TYH media for 10 and 120 min.

**
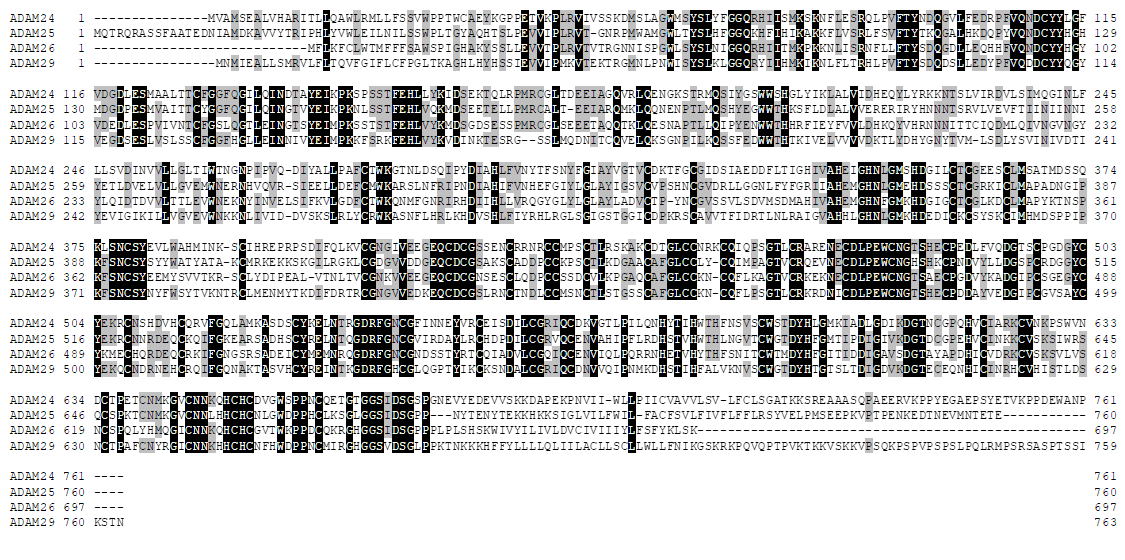
**

**Figure S5. Sequence alignment of murine ADAM24, ADAM25, ADAM26, and ADAM29.** The amino acid residues highlighted in black are conserved among all four proteins, whereas the residues in gray are conserved in three proteins.
