# Supplementary material for: CRISPR/Cas9‐mediated genome editing reveals seven testis‐enriched transmembrane glycoproteins dispensable for male fertility in mice
Source: Andrology. 2023 Dec 12;13(5):1251–60. doi: 10.1111/andr.13564 (PMC11166886; doi:10.1111/andr.13564)
Supplement: Supplementary file 2 — Supporting Information [file ANDR-13-1251-s001.docx]

## Supplementary Tables

**Table S1.** **Mouse lines generated in this study.** These seven mouse lines have been deposited as frozen sperm to RIKEN BioResource Research Center (RBRC) and Center for Animal Resources and Development (CARD) at Kumamoto University.

| **Gene symbol** | **Gene name** | **RBRC No.** | **CARD ID** |
| --- | --- | --- | --- |
| ***4932438H23Rik*** | RIKEN cDNA 4932438H23 gene | 11963 | 3332 |
| ***Adam29*** | A disintegrin and metallopeptidase domain 29 | 11997 | 3366 |
| ***Saysd1*** | SAYSVFN motif domain containing 1 | 11957 | 3326 |
| ***Sel1l2*** | Sel-1 suppressor of lin-12-like 2 (*C. elegans*) | 11961 | 3330 |
| ***Tex2*** | Testis expressed gene 2 | 11971 | 3340 |
| ***Tmigd3*** | Transmembrane and immunoglobulin domain containing 3 | 11960 | 3329 |
| ***Znrf4*** | Zinc and ring finger 4 | 11962 | 3331 |

**Table S2. The sequences of sgRNAs and mutant alleles.** The mutant sequences are displayed as the 20-bp sequences upstream (Up) and downstream (Down) of the truncated genomic regions. Bases in uppercase and lowercase indicate exon and intron sequences, respectively.

| **Gene symbol** | **sgRNAs** | **Mutant sequence** | **Deletion size (bp)** |
| --- | --- | --- | --- |
| ***4932438H23Rik*** | 5’: GCGCTAGGCATCGTTGCCCT  3’: TGTGGCTTTGTTGTAAACGG | Up: CACTTCACAAAAGCTCAGAA  Down: aacaaagccacacctaagag | -678  +5 (GTTGT) |
| ***Adam29*** | 5’: AGTGTTCACCTACTCTGATC  3’: AAGGAAATGCTCAAGGTAGT | Up: ACCTTCCAGTGTTCACCTAC  Down: cttgagcatttccttaggtt | -2442 |
| ***Saysd1*** | 5’: GCGGTTAGCCGAGTTCCGGG  3’: GTTCCTCCTATCCTTGAACG | Up: CGAGCGGGCGGCTGCGCACC  Down: GTGGACTTTAGAGGACAAAG | -5802 |
| ***Sel1l2*** | 5’: AGAGATATTGATCATTATCG  3’: CTTGGCATGAACCTATAAGT | Up: GGCTTTGTTAGTAGAGATAT  Down: CCTCACCAAATAAAGAATTT | -159716 |
| ***Tex2*** | 5’: CCTTTGCACGTGCACTTTGG  3’: TGCATCTGGTGACTAGTATC | Up: GCCAAAACCATCAGCCCCCA  Down: TCTGGTTTGTCTGATGCAAA | -65130 |
| ***Tmigd3*** | 5’: CGGCAGGAATGATTAGAGCG  3’: AGTAGAATATATATGACGAC | Up: ttttcctcaagCAGATCCAG  Down: tggtctgtgtcttggagact | -9878 |
| ***Znrf4*** | 5’: GGTCCACGCAAACCGCGCCA  3’: CGCCCTTCCCGCGTCAGTTG | Up: CTCGGGAGCCGGAGGTAGGG  Down: GGTGCCCAATAAAGCGTGTT | -1036 |

**Table S3. Primers and PCR conditions used for genotyping each mutant mouse line.**

| **Gene symbol** | **Primer sequences** | | **Annealing condition** | **Elongation condition** |
| --- | --- | --- | --- | --- |
|  | WT allele | KO allele |  |  |
| ***4932438H23Rik*** | Fw1: GGGATGTTAACCTTGCTGTC  Rv1: ATCCATACTAAGGCCAGGCA | Fw1:  GGGATGTTAACCTTGCTGTC  Rv2:  GTACTAACCCTATGCATACT | 55 °C  30 s | 72 °C  30 s |
| ***Adam29*** | Fw1: TTCCATAGAAGTGGTGATTCCCATG  Rv1: ATCTCTAATAGTCCATGAAAGCCT | Fw1:  TTCCATAGAAGTGGTGATTCCCATG  Rv2:  ATTGTCTAATGCCAAGTGCTCAGTA | 60 °C  10 s | 68 °C  30 s |
| ***Saysd1*** | Fw1: ACCCTTTCTTTATCTAGTGG  Rv1: ACACCCAGGATATCTGGCTC | Fw1: ACCCTTTCTTTATCTAGTGG  Rv2: GCACATTGGGGCTCCTTTTA | 65 °C  30 s | 72 °C  30 s |
| ***Sel1l2*** | Fw1: TAAGTTTAGCCTTCGTCTCC  Rv1: GTAGAGCTATTTAGGCCAGA | Fw1: TAAGTTTAGCCTTCGTCTCC  Rv2: TGATCTAAGTCCTGACCTCA | 65 °C  30 s | 72 °C  30 s |
| ***Tex2*** | Fw1: AGAAAGCTGGTGTGTGGTTT  Rv1: TAAGTCCAGTGCCATTGGCT | Fw1: AGAAAGCTGGTGTGTGGTTT  Rv2: TAACCTGTGTGGAGTGTGGT | 65 °C  30 s | 72 °C  30 s |
| ***Tmigd3*** | Fw1: GTGTATATGTCAAAGCGGGT  Rv1: GAGCTGTGGGGAACTTGAAA | Fw1: GTGTATATGTCAAAGCGGGT  Rv2: CAGGTATGACTGACATCTCC | 65 °C  30 s | 72 °C  30 s |
| ***Znrf4*** | Fw1: AACTGACATCAAATGAGGCG  Rv1: AGTGTCACCCTGTAGTGGTT | Fw1: AACTGACATCAAATGAGGCG  Rv2: AGTCTCGTTTTCAACTCCCA | 65 °C  30 s | 72 °C  30 s |
|  |  |  |  |  |
